# How to Decide the Iodine Content in Salt for a Country—China as an Example

**DOI:** 10.3390/nu14214606

**Published:** 2022-11-01

**Authors:** Lijun Fan, Yang Du, Fangang Meng, Lixiang Liu, Ming Li, Peng Liu, Dianjun Sun

**Affiliations:** 1 Endemic Disease Control Center, Chinese Center for Disease Control and Prevention, Harbin Medical University, Harbin 150086, China; 2 Key Laboratory of Etiology and Epidemiology (23618504), Education Bureau of Heilongjiang Province, National Health Commission, Harbin 150086, China; 3 Heilongjiang Provincial Key Laboratory of Trace Elements and Human Health, Harbin Medical University, Harbin 150086, China

**Keywords:** iodine deficiency disorders, iodine content, iodized salt, urinary iodine, goiter

## Abstract

Globally, many countries have implemented universal salt iodization to prevent and control iodine deficiency disorders. Therefore, it is important to determine the optimal iodine content in salt and to adjust it in a timely manner. This article aims to establish a process for selecting, deciding, and evaluating the iodine content in salt for China and, if possible, providing references for other countries. Information on salt intake, water iodine, and iodine stability in salt was collected. A field investigation was carried out in different populations in four provinces. Iodine intake was calculated and the appropriate iodine content for salt preliminarily obtained, then verified for suitability with 2020 China National Iodine Deficiency Disorders Surveillance data. In total, 2837 children, 1660 adults, and 2145 pregnant women were enrolled, and their iodine intake from food was 3.7–96.1, 60.0–156.0 and 65.0–112.0 µg/d, respectively. After calculation, when the iodine content in salt was 20, 25 and 30 mg/kg, for children and adults, the total iodine intake ranged from 173.4 to 253.5 µg/d and 230.3 to 379.8 µg/d, respectively. When the iodine content in salt was 30 and 35 mg/kg, for pregnant women, the total iodine intake was 296.8–408.9 µg/d, which was between the recommended nutrient intake and tolerable upper intake level. Therefore, in China, the iodine content in salt in the general population can be preset as 20, 25 and 30 mg/kg, and that in pregnant women 30 and 35 mg/kg, with a variation of ±5 mg/kg based on the automatic spraying technique used in the salt processing plant. Iodine nutritional status was then evaluated according to the preset iodine content in the salt, and it reached the appropriate level for the different populations. The iodine content in salt in China was decided and verified, and the procedure of selecting the iodine content in salt was established for the reference of different countries.

## 1. Introduction

Iodine is an element necessary for the production of thyroid hormones and plays a major role in the growth and development of tissue [1]. A lack of iodine can lead to a spectrum of growth, developmental, and functional morbidities across the whole life span, which are known as iodine deficiency disorders [2]. The consequences of severe iodine deficiency are not only endemic goiter, but also hypothyroidism, cretinism, and decreased fertility rates, etc. [3]. Owing to the high prevalence of iodine deficiency disorders worldwide, ensuring appropriate iodine nutrition for the population and eliminating iodine deficiency disorders is vital for almost all countries.

In 1833, Boussingault initially proposed adding iodine to salt to control endemic goiter. In the 1920s, Kimball used iodized salt for the prevention and treatment of thyroid enlargement in Michigan, USA [4]. One century later, salt iodization had been introduced in many countries worldwide as a sustainable strategy to improve iodine intake and prevent iodine deficiency disorders. In 2021, 124 countries had legislated mandatory salt iodization and at least 21 countries had legislation allowing voluntary salt iodization [4]. The WHO has recommended that household salt be fortified with iodine as a safe and effective strategy for the prevention and control of iodine deficiency disorders, and has also suggested the content for the fortification of food-grade salt with iodine according to estimated salt consumption [5]. However, optimal iodine content in salt is difficult to determine, as it can be affected by many confounding factors, such as the variation in the iodine content in salt during spraying, iodine loss in salt, salt consumption, and iodine concentration in water [4,6,7]. Therefore, these factors should be considered when determining the iodine content in salt for a country. Some countries have found unsuitable levels of iodine content in their salt, measuring close to 100 mg/kg, with excess iodine levels in their residents, including Cameroon, Honduras, and Colombia. Some countries had excess iodine as a result of high water iodine, such as Djibouti, and in other countries it was due to an iodine-rich food intake, such as the Republic of Korea [4].

In China in the 1970s, iodine deficiency disorders were a severe public health problem, and there were 35 million patients with endemic goiter and 250,000 patients with typical endemic cretinism [8]. In 1990, universal salt iodization was agreed to be the best way for iodine deficiency disorder prevention and control [9], and in 1995, it was implemented throughout the country. Thereafter, four adjustments to the iodine content in salt have been made. First, in 1995, the iodine content in salt was required to be above 50, 40, 30 and 20 mg/kg in production, wholesale, retail, and household products, respectively [10]; second, in view of the iodine content in salt without an upper limit [11], in 1997 the upper limit of iodine content in salt was stipulated as <60 mg/kg at the factory level; third, in 1999, due to a high median urinary iodine concentration in children (>300 µg/L), the iodine content in salt was lowered from <60 mg/kg to 35 ± 15 mg/kg in production [12]; and most recently, in 2011, the iodine content in salt was stipulated to have an average concentration of 20, 25 and 30 mg/kg, and the allowable variation range of iodine content in salt was ±30% of the average level. Each province could select one or two iodine contents for their salt from 20, 25 and 30 mg/kg [13]. Along with these four adjustments, the iodine nutrition level of Chinese residents has gradually improved [11]. In 2020, the median urinary iodine concentration was 221.0 µg/L for children aged 8–10 years, and 175.5 µg/L for pregnant women, both at adequate levels [14].

Although iodine status is appropriate for children and pregnant women in China, issues still exist. The first issue is the nearly identical iodine content in salt detected across the provinces, although each province selected different standards for iodine content in salt. A wide variation range of 30% allows salt with the same iodine content to be suitable for all provinces. The second issue is that in some areas, when iodine nutrition status in children is adequate, it is deficient in pregnant women. Therefore, whether pregnant women and children should be supplied with different iodine content in salt needs full consideration.

In this study, China National Iodine Deficiency Disorders Surveillance data were analyzed and related information collected to establish a procedure for determining, evaluating, and selecting appropriate iodine content in salt. Suggestions are given for other countries that have a situation similar to China’s, which can assist in determining optimal iodine content in salt, where appropriate.

## 2. Materials and Methods

**Figure 1:**
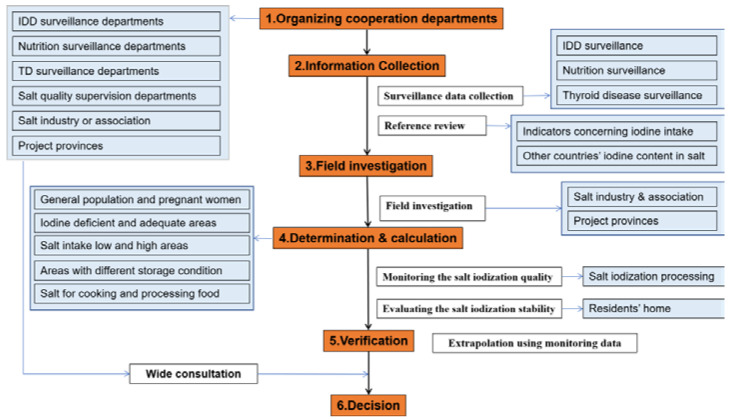
Procedure for selecting the iodine content in salt. IDD, iodine deficiency disorders; TD, thyroid disease.

The Ethics Committee of Harbin Medical University provided approval for this study (HRBMUCEDC20201001). It was conducted according to the provisions of the Declaration of Helsinki. Written informed consent was obtained from all participants or their guardians. The selection process for determining the iodine content in salt is described in Figure 1.

### 2.1. Organizing Cooperating Departments

To accurately select the iodine content in salt, several related government organizations were convened to form a working group, including the Iodine Deficiency Disorders Surveillance Department, Nutrition Surveillance Department, and Salt Quality Supervision Department. The Salt Industry Association and field-investigation provinces were also involved in the project. In China, the National Center for Endemic Disease Control and Prevention was taken as the leading organization of this group.

### 2.2. Information Collection

**Figure 2:**
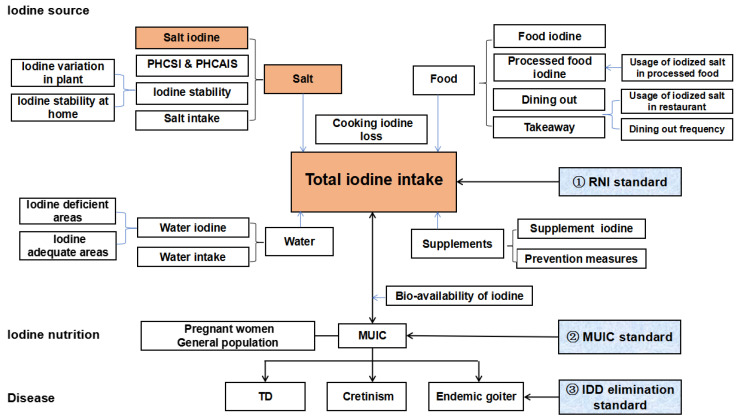
Indicator system for selecting the iodine content in salt. PHCSI, proportion of households consuming iodized salt; PHCAIS, proportion of households consuming adequate iodized salt; RNI, recommended nutrient intake; MUIC, median of urine iodine concentration; IDD, iodine deficiency disorder; TD, thyroid disease.

All data needed for iodine content in salt selection are described in Figure 2. The data were collected from surveillance reports [14,15]. Some parameters for calculation and detection were in accordance with recently published references, with Chinese residents as subjects [16,17,18].

### 2.3. Field Investigation

Because of the large amount of detailed information needed, investigating all provinces of the country was not practical. Therefore, a multistage sampling method was used in this study. According to the geographical distribution of China (east, south, west, and north), four typical provinces were selected—Shanxi, Yunnan, Fujian, and Xinjiang—and one city and one rural county were chosen in each province to balance the possible difference between them. At least 200 children, 100 adults, and 50 pregnant women were enrolled at each site. The inclusion criteria were that participants had lived in the local area for at least 5 years and the age of the participants was 8–10 years for children and 18–45 years for adults and pregnant women. Excluded were participants who could not complete the corresponding survey. Iodine intake was assessed by the Food Frequency Questionnaire specific for iodine-containing foods [16], which is a suitable and valid method for evaluating iodine intake in different populations, including adults, pregnant women and children. Urine samples, drinking water samples, and household salt samples were collected. These were analyzed by the corresponding detection methods [17,18,19]. Statistical analyses were performed using SAS 9.1. Continuous variables with normal distribution are expressed as means ± standard deviation, and those with skewed distribution are expressed as medians and 25th–75th percentiles.

### 2.4. Calculation

**Figure 3:**
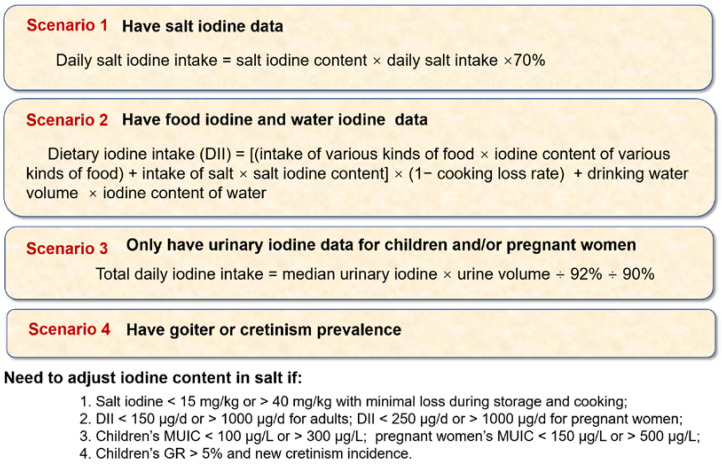
Selection plans for the iodine content in salt for different countries. GR, goiter rate; MUIC, median of urine iodine concentration.

The average drinking water volume of Chinese adults, pregnant women and children were 2, 2 and 1 L [20]. Iodine-deficient areas are areas with water iodine concentrations <40 µg/L, and iodine-adequate areas are areas with water iodine concentrations of 40–100 µg/L [21]. The dietary iodine intake in adults was calculated based on the field investigation. The total daily iodine intake, daily salt iodine intake and daily dietary iodine intake were calculated according to the equations shown in Figure 3.

### 2.5. Decision on the Iodine Content in Salt

Before the iodine content in salt was decided, several questions were considered, including whether different iodine content in salt levels should be offered in different areas (iodine deficient and adequate areas), in diverse populations, and in dissimilar salt intake areas, etc. The variation in the iodine content in salt was considered based on the technology conditions and equipment of the salt plants used for salt iodization, the stability of the iodine in salt, and the determination errors controlled by the laboratory. Full discussion and wide consultation were necessary among the different departments. In accordance with these considerations, the iodine content in salt was preliminarily proposed.

### 2.6. Verification and Evaluation

To evaluate whether the proposed iodine content in salt was suitable for different populations in different areas, further validation was required. Three standards of evaluation were used: dietary reference intakes (DRIs), median urinary iodine concentrations, and iodine deficiency-disorder elimination [22].

For DRIs, a total daily iodine intake between the recommended nutrient intake (RNI) and the tolerable upper intake level (UL) indicated an adequate iodine status. In China, the RNI to UL for children aged 8–10 years is 90–300 µg/d, for pregnant women it is 230–600 µg/d, and for adults it is 120–600 µg/d [23]. In this study, the DRIs of iodine were estimated based on the investigation data from the four provinces.

Sufficient median levels of urinary iodine concentration were 100–299, 150–249 and 100–299 µg/L for children, pregnant women and adults, respectively. The average urine volume of Chinese adults was 1447 mL [24], of pregnant women was 800–2000 mL, and of 8-year-old children was 600–1000 mL. In the calculation process, the average daily urine volume was calculated as 0.8 L for children and 1.4 L for adults and pregnant women. The bioavailability of iodine was calculated as 92%. About 90% of the daily intake of iodine is discharged in urine, and the loss of salt in cooking was calculated to be 20% [25]. The median urinary iodine concentration reported in 2020 China National Iodine Deficiency Disorders Surveillance data was used to estimate the total iodine intake, and then, based on daily salt consumption, the amount of iodine intake from salt was removed, and the remainder was the amount of iodine intake from other sources. Furthermore, the iodine intake from other sources plus the iodine intake from salt, based on the new iodine content in salt, was estimated as the total iodine intake. As 90% of total iodine intake is excreted through urine, it was possible to recalculate a new median urinary iodine concentration.

For iodine deficiency-disorder elimination standards [10], the goiter rate for children and the thyroid disease prevalence in adults are used as long-term indicators, which will be assessed for several months and years after standards for the new iodine content in salt are implemented.

## 3. Results

### 3.1. Information Collection

The iodine-related survey results from China are summarized and presented in Table S1. Other information follows:

Salt: In China, investigation of the salt industry found that the variation in iodine content in salt at processing plants was ±2 mg/kg based on automatic spray machines. Moreover, the grain size and specific surface area of the salt affected the iodization uniformity. The larger the grain size, the larger the variation in the salt’s iodine content. Many factors, including types of salt and iodine fortifier, affect iodine stability in salt [26,27]. In China, the variation of determination was ±2 mg/kg. Currently, the WHO recommends anticipating a 30% loss in the iodization level in the salt from production to consumption [28]. However, in China, as salt in households was in small packages with little loss, only sea salt iodized with potassium iodide was observed to have a 14% loss after nine months. As a result, iodine loss was not taken into account when determining the iodine content in salt. Considering these factors, the allowable range of iodine content in salt was set as 5 mg/kg in China.

Food: The iodine content in prepackaged foods (industrial processed food) varied across different categories. Meat (34.9 µg/100 g) and pickled food (40.5 µg/100 g) had high iodine levels mostly due to processing with iodized salt [29]. The iodine content in different condiments varied, and varied even in the same kind of condiment from different manufacturers [30]. In this study, the iodine content was 23.2 mg/kg in edible salt, and an average 3.0 µg/100 g in 28 kinds of soy sauce. In China, the iodine in these types of food was taken into account in the calculation of dietary iodine.

Water and supplements: During 2017–2018, the National Water Iodine Surveillance results showed the median water iodine concentration was 3.4 µg/L at the township level, and 95% of townships had a water iodine concentration <40 µg/L, nearly 3% had a concentration of 40–100 µg/L and 2% had >100 µg/L, which identified China as an iodine-deficient country. In iodine-deficient areas, assuming 1, 2 and 2 L as the drinking water volume for children, adults and pregnant women, respectively, the iodine intake from water was 0–40, 0–80 and 0–80 µg iodine per day, and in iodine-adequate areas, the intake was 40–100, 80–200 and 80–200 µg/d. In terms of iodine supplements, according to 2020 China National Iodine Deficiency Disorders Surveillance data, few people used iodine preparations in China (i.e., 3.3% respondents were taking iodine preparations). Iodized salt was the main method of supplementing iodine; therefore, this factor was not taken into account when setting the standard for iodine content in salt.

### 3.2. Field Investigation

**Figure 4:**
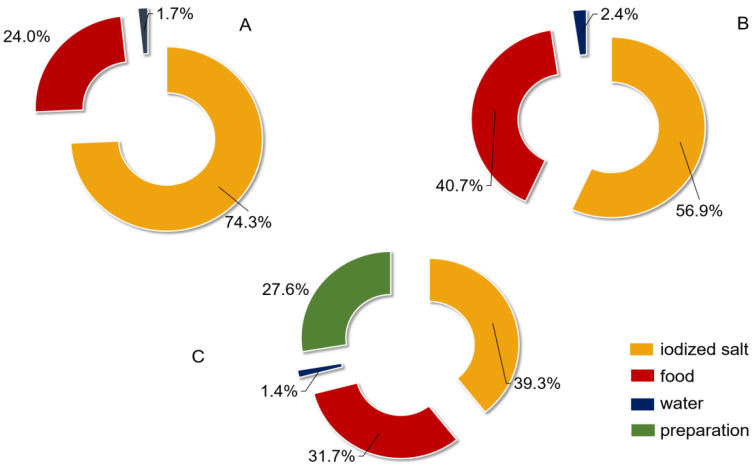
Contribution of iodine from different sources for different populations. (**A**) children; (**B**) adults; (**C**) pregnant women.

A total of 2837 children, 1660 adults and 2145 pregnant women were investigated and their basic information obtained. It was determined that they were all iodine-adequate or -sufficient (Table 1). The iodine intake from food was 3.7–96.1 µg/d in children, 60.0–156.0 µg/d in adults and 65.0–112.0 µg/d in pregnant women. Salt intake was 6.0–8.5 g/d and 4.8–8.7 g/d in adults and in pregnant women, respectively. Regarding the contribution of different iodine sources to the total iodine intake, in children, the contribution of iodized salt was considerably higher than that in adults and pregnant women, and in adults and pregnant women, the food-source iodine was higher than that in children, besides, pregnant women did take a significant portion (27.6%) of their iodine intake from preparations (Table 1, Figure 4).

### 3.3. Determination and Verification

Based on the collected information and the field investigation in the four provinces, the food iodine intake, salt iodine intake and total iodine intake were calculated with different iodine contents in salt (Table 2). From these results, a tentative iodine content in salt of 20, 25 and 30 mg/kg for the general population and 30 and 35 mg/kg for pregnant women was set forth. Evaluation through standards of RNI and the median urinary iodine concentration would be followed to ensure the suitability of the iodine content in salt.

The results of the evaluation by RNI are described in Table 3 and Tables S2–S4. The verification results showed that the three ranges of iodine content in salt for the general population (including children and adults) and two ranges for pregnant women could basically meet the iodine nutritional needs in iodine-deficient areas and iodine-adequate areas with different salt intakes (basically between RNI and UL).

**Figure 5:**
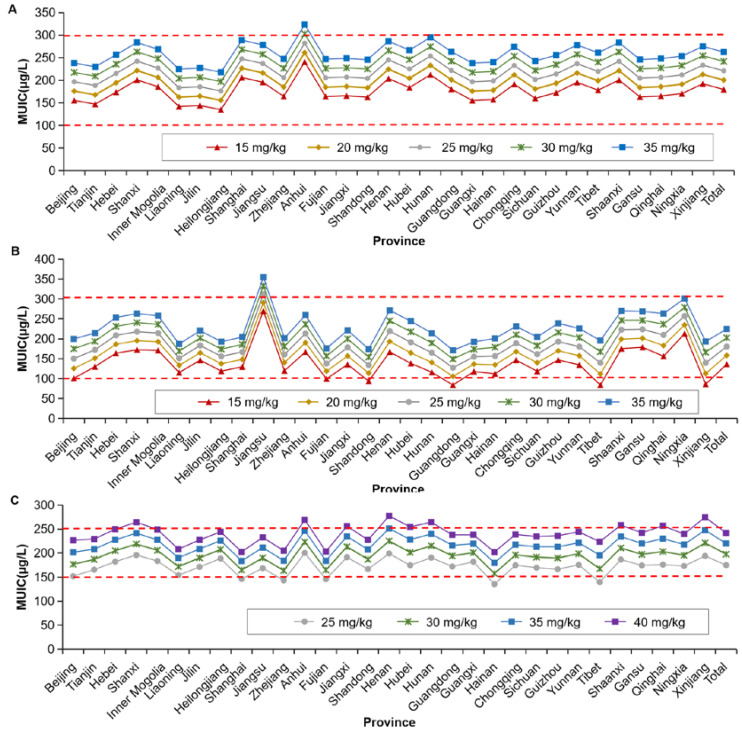
Evaluation by median urinary iodine concentration standard for children, adults and pregnant women under condition of the presumed different iodine content in salt. (**A**) for children; (**B**) for adults; (**C**) pregnant women. When the iodine content in salt was set as 20 mg/kg (varied from 15 to 25 mg/kg), 25 mg/kg (varied from 20 to 30 mg/kg) or 30 mg/kg (varied from 25 to 35 mg/kg), MUIC in children and adults could reach the sufficient levels. When the iodine content in salt was set as 30 mg/kg (varied from 25 to 35 mg/kg) or 35 mg/kg (varied from 30 to 35 mg/kg), MUIC in pregnant women reached sufficient levels. MUIC, median of urine iodine concentration.

The data were evaluated according to median urinary iodine concentration. Based on the salt consumption in each province and the urine iodine concentration in 2020 China National Iodine Deficiency Disorders Surveillance data, each province was able to select their own iodine content in salt from the preset iodine content, and for the general population, when different iodine contents in salt were taken (20, 25, 30 mg/kg, plus lower and upper variation limits, 15 and 35 mg/kg), the median urinary iodine concentrations reached sufficient levels; and for pregnant women, when different iodine contents in salt were taken (2 contents, 30, 35 mg/kg, plus lower and upper variation limits, 25 and 40 mg/kg), the median urinary iodine concentrations reached sufficient levels (Figure 5).

Further evaluation of the elimination of iodine deficiency disorders will be carried out in the future to ensure the suitability of the iodine content in salt.

Based on each country’s surveillance data, different plans for managing iodine content in salt can be proposed (Figure 3). However, iodine content in salt should not be lower or higher than the recommended range of 15–40 mg/kg if iodine loss is reasonable. Otherwise, iodine deficiency or excess will emerge [30].

## 4. Discussion

Globally, universal salt iodization policies have been implemented in 145 countries [4]; however, not all countries with universal salt iodization policies have achieved adequate levels of iodine nutrition in their population [31]. According to data collected by the Iodine Global Network (IGN), currently 21 countries or areas (without universal salt iodization or low-iodized salt) are iodine-deficient and 13 countries or areas experience iodine excess (either due to excess groundwater iodine, overiodized salt or overconsumed iodine-rich food) [3]. Both iodine deficiency and iodine excess increase the prevalence of thyroid diseases, especially iodine deficiency. Even mild-to-moderate iodine deficiency can impair health [32,33]. In mild-to-moderate iodine deficiency, increased thyroid activity can compensate for low iodine intake and maintain euthyroidism in most individuals, but chronic thyroid stimulation increases the prevalence of toxic nodular goiters and hyperthyroidism in the population [34,35]. This indicates that the selection of appropriate iodine content is important in maintaining adequate iodine nutrition levels. This research collected data related to Chinese iodine deficiency disorders and carried out field investigations to determine the iodine content in salt and evaluate its suitability.

Before the final decision was made, several issues were addressed: (1) whether water iodine concentration varies in different areas; (2) which population should be considered when defining the iodine content in salt; (3) whether salt intake varies among different areas; (4) whether the iodized salt in processed food should be considered; and (5) assessing the stability of iodine additives: potassium iodide, potassium iodate and seaweed iodine.

First, the water iodine concentration has a large impact on iodine nutrition. Recently, China’s newly issued standard [21] stipulated the iodine-adequate areas (water iodine concentration in the range 40–100 µg/L) and iodine-deficient areas (water iodine concentration < 40 µg/L). In iodine-deficient areas, it is necessary to consume qualified iodized salt to ensure an adequate iodine nutritional level. In waterborne iodine-excess areas, noniodized salt should be supplied to alleviate the iodine excess damage, while in adequate areas, because the population’s total iodine intake is between RNI and UL and is in a safe range, either salt can be supplied (iodized or noniodized) or both can be supplied simultaneously. As there are also some countries that have high concentrations of iodine in water, such as Djibouti and Qatar, this suggestion might be applicable.

Second, from the 2020 China National Iodine Deficiency Disorders Surveillance results, at provincial level, the iodine nutrition levels of children in all provinces in China were at a sufficient level (100–300 µg/L), but iodine nutrition of pregnant women in some provinces was mildly deficient (100–150 µg/L). At a county level, the iodine nutrition levels in children in 94.0% of the monitoring counties were adequate, while the levels in pregnant women in 73.1% of the monitoring counties were adequate. In addition, through years of monitoring, it was found that the correlation coefficient between the urinary iodine of school-age children and pregnant women was low (about 0.3) [36]. Therefore, iodine nutrition levels in school-aged children should not be taken as proxy for iodine nutrition levels in pregnant women. When the salt intake of the general population and pregnant women is the same and the iodine nutrition of children is appropriate, then pregnant women will be in a state of mild deficiency. When iodine nutrition is appropriate for pregnant women, children may be at risk of iodine overdose. Therefore, it is necessary to set differentiated iodine contents in salt for the general population and pregnant women. Globally, only Papua New Guinea was found to have two standards for iodine content: one for cooking salt and the other for table salt.

Third, the salt intake of Chinese residents has been decreasing from 1991 to 2018 [25]; however, the amount still exceeded recommendations of the WHO (<5 g/d). A study in 2019 showed mean daily salt intake ranged from 6.75 g/d to 10.66 g/d in 13 countries, including the United States, Samoa, and Fiji [37]. Compared with these countries, salt intake of Chinese residents is still high. Cooking salt is one source of sodium, and sodium intake in China is also among the highest in the world [38]. The amount of salt intake is related to the iodine content in salt. Therefore, when revising the standard for iodine content in salt, the salt intake of residents in different areas should be considered. In 2013, the WHO published public health strategies for salt reduction and iodine fortification with the aim of a 30% reduction in salt intake [39]. Thereafter, concerns emerged about the possibility of conflict between salt intake reduction and iodine supplementation [40]. The Chinese State Council implemented the Healthy China Action, which stipulated that China would implement a national diet campaign and encourage the whole of society to reduce salt, oil and sugar [41]. In the future, the influence of dietary salt reduction on its contribution to the iodine intake should be reconsidered when deciding the iodine content in salt.

Fourth, although universal salt iodization calls for fortification with iodine in all food-grade salt, in practice, besides the table salt that was fortified, some salt for the food industry or animal consumption was not fortified. In developed countries, except iodized salt, daily iodine intake also comes from foods such as milk and dairy products, eggs, seafood and processed food [42,43]. These foods can be divided into two categories, naturally iodine-rich food and processed food with iodized salt. In middle- and higher-income countries, most of the daily salt intake comes from processed foods instead of cooking and table salt, which makes iodine content in salt seem of low importance. However, if most of the processed food were processed using cooking salt with the same iodine content as table salt, then the only difference would be the amount of salt added during processing and home cooking. If the processed food used noniodized salt, it would have a great influence on health. Nevertheless, in many low- and middle-income countries, discretionary salt (cooking and table) continues to be the main source of salt consumption [44]. In China, although nearly 90% of processed food contains iodized salt, because the amount of processed food consumed is small, cooking salt is still the main source of iodine intake. Overall, different countries have different dietary customs. It is recommended that a region-specific iodine food-frequency questionnaire be established, and measures should be adjusted accordingly.

This research recorded the whole process for determining iodine content in salt, and comprehensively analyzed all aspects influencing the iodine content in salt. A limitation of this study is that the contribution of iodine from restaurant food and beverages was not investigated. In the future, the effects of the proposed iodine content in salt must be observed and evaluated according to the iodine deficiency-disorder elimination standards.

## Figures and Tables

**Table 1:** Iodine supplementation and its contribution in children, adults and pregnant women.

Population	District	*N*	MUIC (μg/L) Median, P25−P75	Salt Iodine (mg/kg) $\bar{x}$ ± *s*	Consumption Rate (%)			Contribution Proportion (%)			
					Salt ^1^	Iodine Preparations	Iodine−Rich Food	Salt ^1^	Food	Water	Iodine Preparations
Children	Fujian	814	213.7, 136.0−309.6	21.7 ± 7.8	86.1	−	80.4	60.5	38.2	1.3	−
	Shanxi	819	234.5, 129.1−382.5	25.2 ± 4.9	93.3	−	65.5	73.9	24.0	2.1	−
	Yunnan	400	250.6, 179.2−323.4	25.4 ± 3.7	96.0	−	−	56.8	40.6	2.6	−
	Xinjiang	804	258.0, 179.3−332.0	26.9 ± 4.0	92.9	−	77.9	97.3	1.2	1.4	−
	Total	2837	238.2, 154.1−335.5	24.8 ± 8.0	91.5	−	74.6	74.3	24.0	1.7	−
Adults	Fujian	376	168.7, 103.6−228.7	24.6 ± 2.7	89.7	0.3	53.2	57.0	41.2	1.8	−
	Shanxi	345	180.3, 104.1−259.9	25.6 ± 4.9	92.5	2.0	23.3	67.8	27.0	5.2	−
	Yunnan	388	186.7, 118.6−253.7	25.8 ± 2.8	98.5	0.7	37.0	64.5	32.7	2.8	−
	Xinjiang	384	224.3, 165.6−282.1	28.2 ± 5.3	94.5	0.5	7.8	42.6	56.8	0.6	−
	Total	1493	187.3, 122.8−258.1	26.1 ± 4.3	93.9	0.8	29.7	56.9	40.7	2.4	−
Pregnantwomen	Fujian	338	145.4, 100.5−214.7	23.8 ± 5.4	100.0	10.7	66.6	50.6	41.1	1.0	7.3
	Shanxi	874	161.5, 91.2−247.2	25.3 ± 5.4	96.6	2.0	32.5	53.3	43.3	2.7	0.7
	Yunnan	413	138.7, 91.9−192.8	26.0 ± 2.8	98.0	1.0	32.2	59.5	38.8	1.7	−
	Xinjiang ^2^	503	216.2, 145.7−288.8	27.2 ± 4.1	78.4	7.6	20.8	20.6	20.2	1.5	57.7
	Total	2128	164.5, 104.8−243.0	25.7 ± 4.8	93.4	5.2	35.5	39.3	31.7	1.4	27.6

Note: ^1^ Qualified iodized salt; MUIC, median of urine iodine concentration. ^2^ In Xinjiang, iodized oil capsules were taken as a government measure for preventing cretinism.

**Table 2:** Iodine intake of children, adults and pregnant women from different iodine sources in different water iodine areas.

Iodine Content in Salt (mg/kg)	Children (μg/d)				Adults (μg/d)				Pregnant Women (μg/d)			
	Water ^1^	Food	Salt ^2^	Total	Water	Food	Salt	Total	Water	Food	Salt	Total
Iodine-deficient areas												
15	0–40	3.7–96.1	75.0	63.0–192.9	0–80	60–156	127.5	150.0–306.8	0–80	65–112	130.5	156.4–274.0
20	0–40	3.7–96.1	100.0	83.0–212.9	0–80	60–156	170.0	184.0–340.8	0–80	65–112	174.0	191.2–308.8
25	0–40	3.7–96.1	125.0	103.0–232.9	0–80	60–156	212.5	218.0–374.8	0–80	65–112	217.5	226.0–343.6
30	0–40	3.7–96.1	150.0	123.0–252.9	0–80	60–156	255.0	252.0–408.8	0–80	65–112	261.0	260.8–378.4
35	0–40	3.7–96.1	175.0	143.0–272.9	0–80	60–156	297.5	286.0–442.8	0–80	65–112	304.5	295.6–413.2
40	0–40	3.7–96.1	200.0	163.0–292.9	0–80	60–156	340.0	320.0–476.8	0–80	65–112	348.0	330.4–448.0
45	0–40	3.7–96.1	225.0	183.0–312.9	0–80	60–156	382.5	354.0–510.8	0–80	65–112	391.5	365.2–482.8
Iodine-adequate areas												
15	40–100	3.7–96.1	75.0	119.0–276.9	80–200	60–156	127.5	230.0–426.8	80–200	65–112	130.5	236.4–394.0
20	40–100	3.7–96.1	100.0	139.0–296.9	80–200	60–156	170.0	264.0–460.8	80–200	65–112	174.0	271.2–428.8
25	40–100	3.7–96.1	125.0	159.0–316.9	80–200	60–156	212.5	298.0–494.8	80–200	65–112	217.5	306.0–463.6
30	40–100	3.7–96.1	150.0	179.0–336.9	80–200	60–156	255.0	332.0–528.8	80–200	65–112	261.0	340.8–498.4
35	40–100	3.7–96.1	175.0	199.0–356.9	80–200	60–156	297.5	366.0–562.8	80–200	65–112	304.5	375.6–533.2
40	40–100	3.7–96.1	200.0	219.0–376.9	80–200	60–156	340.0	400.0–596.8	80–200	65–112	348.0	410.4–568.0
45	40–100	3.7–96.1	225.0	239.0–396.9	80–200	60–156	382.5	434.0–630.8	80–200	65–112	391.5	445.2–602.8

Note: ^1^ Taking 1, 2 and 2 L as drinking water volume for children, adults and pregnant women, in iodine-deficient areas, the iodine intake was 0–40, 0–80 and 0–80 µg iodine per day from water, and for iodine-adequate areas, the intake was 40–100, 80–200 and 80–200 µg/d; ^2^ data were estimated according to the salt consumption levels recommended in the Chinese Dietary Guidelines.

**Table 3:** Estimated iodine intake and urine iodine levels after changes in salt iodine content in China.

Population	Iodine Content in Salt ^1^ (mg/kg)	P-SII ^2^ (μg/d)	P-TII ^3^ (μg/d)	E-MUIC ^4^ (μg/L)
Children	15	60.0	173.5	179.6
	20	80.0	193.5	200.3
	25	100.0	213.5	221.0
	30	120.0	233.5	241.7
	35	140.0	253.5	262.4
Adults	15	112.1	230.3	136.2
	20	149.5	267.7	158.3
	25	186.9	305.0	180.4
	30	224.2	342.4	202.5
	35	261.6	379.8	224.6
Pregnant women	25	186.9	296.8	175.5
	30	224.2	334.1	197.6
	35	261.6	371.5	219.7
	40	299.0	408.9	241.8

Note: ^1^ Salt iodine content; ^2^ estimated daily salt iodine intake according to the latest salt consumption and the preset salt iodine content; ^3^ estimated total daily iodine intake from diet, drinking water and salt according to the preset salt iodine content; ^4^ estimated median urinary iodine after adjustment for salt iodine content.

## Data Availability

Data described in the manuscript, code book, and analytic code will be made available upon the authors’ approval.

## Supplementary Materials

The following supporting information can be downloaded at https://www.mdpi.com/article/10.3390/nu14214606/s1. Table S1: Basic information concerning iodine intake and iodine nutrition in China; Table S2: Estimated iodine intake and urine iodine levels after changes in salt iodine content in 31 provinces of China (children); Table S3: Estimated iodine intake and urine iodine levels after changes in salt iodine content in 31 provinces of China (adults); Table S4: Estimated iodine intake and urine iodine levels after changes in salt iodine content in 31 provinces of China (pregnant women).
