# Supplementary material for: How to Decide the Iodine Content in Salt for a Country—China as an Example
Source: Nutrients. 2022 Nov 1;14(21):4606. doi: 10.3390/nu14214606 (PMC9653880; doi:10.3390/nu14214606)
Supplement: Supplementary file 1 [file nutrients-14-04606-s001.zip › nutrients-1987258-supplementary.pdf]

Table S1. Basic information concerning iodine intake and iodine nutrition in China.

Province	Water iodine (μg/L)	Salt					Iodine supplement in pregnant women (%)	MUIC (μg/L)			Children Goiter rate (%)
		Salt iodine			CRIS (%)	CRQIS (%)		children	Pregnant women	Adults[a]	
		Mean (mg/kg)	SD (mg/kg)	CV (%)							
Beijng	3.4	24.4	4.95	20.3	92.4	86.5	75.5	194.2	149.2	147.2	3.0
Tianjin	5.1	24.8	5.38	21.7	67.0	55.7	1.4	187.6	164.9	171.4	2.5
Hebei	4.7	23.3	3.77	16.2	96.0	91.1	1.5	208.0	174.6	201.1	1.6
Shanxi	5.0	23.8	3.90	16.4	98.7	95.0	0.3	237.2	190.5	212.4	2.1
Inner Mongolia	6.7	23.3	4.54	19.5	98.8	94.2	0.7	220.0	176.3	207.0	1.3
Liaoning	3.7	24.3	3.23	13.3	98.9	97.2	6.9	180.4	151.6	148.6	1.8
Jilin	4.6	24.1	3.90	16.2	99.8	97.3	1.5	181.9	168.1	179.9	0.5
Heilongjiang	5.0	25.0	4.30	17.2	99.5	97.9	0.1	176.3	188.7	155.8	0.5
Shanghai	2.7	24.1	5.21	21.6	65.4	53.6	4.7	243.6	142.9	163.5	2.0
Jiangsu	7.3	23.8	3.67	15.4	98.1	95.1	2.3	232.0	163.8	307.0	1.9
Zhejiang	2.1	23.6	3.82	16.2	81.1	76.3	12.2	199.9	137.6	154.9	2.2
Anhui	4.3	24.0	3.17	13.2	96.3	94.0	0.7	277.9	195.4	208.7	1.3
Fujian	2.2	24.4	2.88	11.8	94.5	92.3	4.8	202.9	143.6	135.4	1.8
Jiangxi	2.5	24.6	3.79	15.4	99.7	96.7	1.3	205.3	189.7	176.7	0.3
Shandong	9.2	23.5	4.75	20.2	85.4	77.7	1.1	197.9	160.8	127.9	2.7
Henan	9.6	26.2	4.27	16.3	98.0	92.6	0.3	250.1	205.1	225.5	1.2
Hubei	3.7	25.2	3.91	15.5	99.5	96.0	1.3	225.9	175.9	192.2	1.3
Hunan	2.5	26.8	4.13	15.4	99.6	95.8	0.6	261.1	199.3	173.8	0.8
Guangdong	5.1	24.9	3.31	13.3	98.2	97.0	0.8	221.3	171.9	127.0	0.8
Guangxi	3.3	24.6	3.74	15.2	98.9	95.9	0.2	194.9	180.6	153.6	0.5
Hainan	6.0	24.0	3.31	13.8	98.2	95.4	0.1	194.5	131.1	152.2	0.1
Chongqing	1.5	26.1	4.20	16.1	99.3	94.6	0.6	237.2	179.6	193.5	2.1
Sichuan	2.1	27.2	4.05	14.9	99.2	95.5	1.9	210.5	179.3	170.9	1.7
Guizhou	1.8	26.6	3.83	14.4	99.9	96.6	0.4	220.5	174.0	200.2	1.3
Yunnan	1.3	24.3	3.94	16.2	99.5	95.9	0.6	233.8	172.5	177.0	1.3
Tibet	2.2	28.2	6.77	24.0	99.7	95.2	19.2	232.7	131.5	148.3	0.1
Shannxi	4.6	24.6	3.67	14.9	99.9	97.1	1.8	240.1	184.7	220.9	1.6

Gansu	2.2	25.9	3.76	14.5	99.5	95.2	0.5	208.2	178.7	227.9	1.1
Qinghai	1.7	26.6	4.34	16.3	98.8	93.5	5.8	213.1	184.8	218.3	0.7
Ningxia	6.4	25.9	4.53	17.5	99.1	94.1	1.0	215.6	177.3	261.0	0.6
Xinjiang	3.8	27.5	4.43	16.1	99.9	96.5	25.4	243.8	207.4	152.9	0.5
Total	3.4	25.0	4.30	17.2	95.9	92.0	3.3	221.0	175.5	180.4	1.5

Note: [a] Data from TIDE project; SD, The standard deviation; CRIS, coverage rate of iodized salt; CRQIS, consumption rate of qualified iodized salt; MUIC, median urine iodine concentration; TD, thyroid disease.

Table S2. Estimated iodine intake and urine iodine levels after changes in salt iodine content in 31 provinces of China (children).

Province	M-TII (μg/d)	M-SII (μg/d)	Iodine content 15 mg/kg			Iodine content 20 mg/kg			Iodine content 25 mg/kg			Iodine content 30 mg/kg			Iodine content 35 mg/kg		
			P-SII (μg/d)	P-TII (μg/d)	E-MUIC (μg/L)	P-SII (μg/d)	P-TII (μg/d)	E-MUIC (μg/L)	P-SII (μg/d)	P-TII (μg/d)	E-MUIC (μg/L)	P-SII (μg/d)	P-TII (μg/d)	E-MUIC (μg/L)	P-SII (μg/d)	P-TII (μg/d)	E-MUIC (μg/L)
Beijing	187.6	97.6	60.0	150.0	155.3	80.0	170.0	176.0	100.0	190.0	196.7	120.0	210.0	217.4	140.0	230.0	238.1
Tianjin	181.3	99.2	60.0	142.1	147.0	80.0	162.1	167.7	100.0	182.1	188.4	120.0	202.1	209.1	140.0	222.1	229.8
Hebei	201.0	93.2	60.0	167.8	173.6	80.0	187.8	194.3	100.0	207.8	215.0	120.0	227.8	235.7	140.0	247.8	256.4
Shanxi	229.2	95.2	60.0	194.0	200.8	80.0	214.0	221.5	100.0	234.0	242.2	120.0	254.0	262.9	140.0	274.0	283.6
Inner Mongolia	212.6	93.2	60.0	179.4	185.6	80.0	199.4	206.3	100.0	219.4	227.0	120.0	239.4	247.7	140.0	259.4	268.4
Liaoning	174.3	97.2	60.0	137.1	141.9	80.0	157.1	162.6	100.0	177.1	183.3	120.0	197.1	204.0	140.0	217.1	224.7
Jilin	175.7	96.4	60.0	139.3	144.2	80.0	159.3	164.9	100.0	179.3	185.6	120.0	199.3	206.3	140.0	219.3	227.0
Heilongjiang	170.3	100.0	60.0	130.3	134.9	80.0	150.3	155.6	100.0	170.3	176.3	120.0	190.3	197.0	140.0	210.3	217.7
Shanghai	235.4	96.4	60.0	199.0	205.9	80.0	219.0	226.6	100.0	239.0	247.3	120.0	259.0	268.0	140.0	279.0	288.7
Jiangsu	224.2	95.2	60.0	189.0	195.6	80.0	209.0	216.3	100.0	229.0	237.0	120.0	249.0	257.7	140.0	269.0	278.4
Zhejiang	193.1	94.4	60.0	158.7	164.3	80.0	178.7	185.0	100.0	198.7	205.7	120.0	218.7	226.4	140.0	238.7	247.1
Anhui	268.5	96.0	60.0	232.5	240.6	80.0	252.5	261.3	100.0	272.5	282.0	120.0	292.5	302.7	140.0	312.5	323.4
Fujian	196.0	97.6	60.0	158.4	164.0	80.0	178.4	184.7	100.0	198.4	205.4	120.0	218.4	226.1	140.0	238.4	246.8
Jiangxi	198.4	98.4	60.0	160.0	165.6	80.0	180.0	186.3	100.0	200.0	207.0	120.0	220.0	227.7	140.0	240.0	248.4
Shandong	191.2	94.0	60.0	157.2	162.7	80.0	177.2	183.4	100.0	197.2	204.1	120.0	217.2	224.8	140.0	237.2	245.5
Henan	241.6	104.8	60.0	196.8	203.7	80.0	216.8	224.4	100.0	236.8	245.1	120.0	256.8	265.8	140.0	276.8	286.5
Hubei	218.3	100.8	60.0	177.5	183.7	80.0	197.5	204.4	100.0	217.5	225.1	120.0	237.5	245.8	140.0	257.5	266.5
Hunan	252.3	107.2	60.0	205.1	212.2	80.0	225.1	232.9	100.0	245.1	253.6	120.0	265.1	274.3	140.0	285.1	295.0
Guangdong	213.8	99.6	60.0	174.2	180.3	80.0	194.2	201.0	100.0	214.2	221.7	120.0	234.2	242.4	140.0	254.2	263.1
Guangxi	188.3	98.4	60.0	149.9	155.2	80.0	169.9	175.9	100.0	189.9	196.6	120.0	209.9	217.3	140.0	229.9	238.0
Hainan	187.9	96.0	60.0	151.9	157.2	80.0	171.9	177.9	100.0	191.9	198.6	120.0	211.9	219.3	140.0	231.9	240.0
Chongqing	229.2	104.4	60.0	184.8	191.2	80.0	204.8	211.9	100.0	224.8	232.6	120.0	244.8	253.3	140.0	264.8	274.0

Province	M-TII (μg/d)	M-SII (μg/d)	Iodine content 15 mg/kg			Iodine content 20 mg/kg			Iodine content 25 mg/kg			Iodine content 30 mg/kg			Iodine content 35 mg/kg		
			P-SII (μg/d)	P-TII (μg/d)	E-MUIC (μg/L)	P-SII (μg/d)	P-TII (μg/d)	E-MUIC (μg/L)	P-SII (μg/d)	P-TII (μg/d)	E-MUIC (μg/L)	P-SII (μg/d)	P-TII (μg/d)	E-MUIC (μg/L)	P-SII (μg/d)	P-TII (μg/d)	E-MUIC (μg/L)
Sichuan	203.4	108.8	60.0	154.6	160.0	80.0	174.6	180.7	100.0	194.6	201.4	120.0	214.6	222.1	140.0	234.6	242.8
Guizhou	213.0	106.4	60.0	166.6	172.5	80.0	186.6	193.2	100.0	206.6	213.9	120.0	226.6	234.6	140.0	246.6	255.3
Yunnan	225.9	97.2	60.0	188.7	195.3	80.0	208.7	216.0	100.0	228.7	236.7	120.0	248.7	257.4	140.0	268.7	278.1
Tibet	224.8	112.8	60.0	172.0	178.1	80.0	192.0	198.8	100.0	212.0	219.5	120.0	232.0	240.2	140.0	252.0	260.9
Shaanxi	232.0	98.4	60.0	193.6	200.4	80.0	213.6	221.1	100.0	233.6	241.8	120.0	253.6	262.5	140.0	273.6	283.2
Gansu	201.2	103.6	60.0	157.6	163.1	80.0	177.6	183.8	100.0	197.6	204.5	120.0	217.6	225.2	140.0	237.6	245.9
Qinghai	205.9	106.4	60.0	159.5	165.1	80.0	179.5	185.8	100.0	199.5	206.5	120.0	219.5	227.2	140.0	239.5	247.9
Ningxia	208.3	103.6	60.0	164.7	170.5	80.0	184.7	191.2	100.0	204.7	211.9	120.0	224.7	232.6	140.0	244.7	253.3
Xinjiang	235.6	110.0	60.0	185.6	192.1	80.0	205.6	212.8	100.0	225.6	233.5	120.0	245.6	254.2	140.0	265.6	274.9
Total	213.5	100.0	60.0	173.5	179.6	80.0	193.5	200.3	100.0	213.5	221.0	120.0	233.5	241.7	140.0	253.5	262.4

Note: M-TII represents the estimated total daily iodine intake from diet, drinking water and salt according to the latest MUIC;
M-SII represents the estimated daily salt iodine intake according to the recommended salt consumption by Chinese Dietary Guidelines and the actual iodine content;
P-SII represents the estimated daily salt iodine intake of each province according to the recommended salt consumption by Chinese Dietary Guidelines and the preset salt iodine content;
P-TII represents the estimated total daily iodine intake from diet, drinking water and salt according to the preset salt iodine content;
E-MUIC represents the estimated median of urinary iodine after the adjustment of salt iodine content.

Table S3. Estimated iodine intake and urine iodine levels after changes in salt iodine content in 31 provinces of China (adults).

Province	M-TII (μg/d)	M-SII (μg/d)	Iodine content 15 mg/kg			Iodine content 20 mg/kg			Iodine content 25 mg/kg			Iodine content 30 mg/kg			Iodine content 35 mg/kg		
			P-SII (μg/d)	P-TII (μg/d)	E-MUIC (μg/L)	P-SII (μg/d)	P-TII (μg/d)	E-MUIC (μg/L)	P-SII (μg/d)	P-TII (μg/d)	E-MUIC (μg/L)	P-SII (μg/d)	P-TII (μg/d)	E-MUIC (μg/L)	P-SII (μg/d)	P-TII (μg/d)	E-MUIC (μg/L)
Beijing	248.9	203.8	125.3	170.4	100.8	167.1	212.1	125.5	208.8	253.9	150.2	250.6	295.7	174.9	292.4	337.4	199.6
Tianjin	289.8	177.1	107.1	219.8	130.0	142.8	255.5	151.1	178.5	291.2	172.2	214.2	326.9	193.4	249.9	362.6	214.5
Hebei	340.0	177.0	114.0	277.0	163.8	152.0	314.9	186.3	190.0	352.9	208.7	227.9	390.9	231.2	265.9	428.9	253.7
Shanxi	359.1	182.5	115.0	291.7	172.5	153.4	330.0	195.2	191.7	368.3	217.8	230.0	406.7	240.5	268.4	445.0	263.2
Inner Mongolia	350.0	171.5	110.4	288.9	170.9	147.2	325.7	192.6	184.1	362.5	214.4	220.9	399.3	236.2	257.7	436.1	257.9
Liaoning	251.3	147.6	91.1	194.8	115.2	121.5	225.1	133.2	151.8	255.5	151.1	182.2	285.9	169.1	212.6	316.2	187.0
Jilin	304.2	151.1	94.1	247.1	146.2	125.4	278.5	164.7	156.8	309.8	183.2	188.1	341.2	201.8	219.5	372.5	220.3
Heilongjiang	263.4	155.9	93.6	201.1	118.9	124.7	232.2	137.4	155.9	263.4	155.8	187.1	294.6	174.2	218.3	325.8	192.7
Shanghai	276.4	152.4	94.8	218.9	129.5	126.4	250.5	148.2	158.0	282.1	166.9	189.7	313.7	185.6	221.3	345.4	204.3
Jiangsu	519.1	170.4	107.4	456.1	269.7	143.2	491.9	290.9	178.9	527.7	312.1	214.7	563.5	333.2	250.5	599.2	354.4
Zhejiang	261.9	163.2	103.7	202.4	119.7	138.3	237.0	140.2	172.9	271.6	160.6	207.4	306.2	181.1	242.0	340.7	201.5
Anhui	352.9	187.0	116.9	282.7	167.2	155.8	321.7	190.3	194.8	360.7	213.3	233.8	399.6	236.4	272.7	438.6	259.4
Fujian	228.9	157.2	96.7	168.4	99.6	128.9	200.6	118.6	161.1	232.8	137.7	193.3	265.0	156.7	225.5	297.2	175.8
Jiangxi	298.8	178.7	109.0	229.0	135.5	145.3	265.4	156.9	181.6	301.7	178.4	217.9	338.0	199.9	254.3	374.3	221.4
Shandong	216.3	159.7	101.9	158.5	93.7	135.9	192.5	113.8	169.9	226.4	133.9	203.8	260.4	154.0	237.8	294.4	174.1
Henan	381.3	231.4	132.5	282.4	167.0	176.6	326.5	193.1	220.8	370.7	219.2	264.9	414.8	245.3	309.1	459.0	271.5
Hubei	325.0	225.7	134.4	233.6	138.2	179.2	278.4	164.7	223.9	323.2	191.1	268.7	368.0	217.6	313.5	412.8	244.1
Hunan	293.9	222.7	124.6	195.8	115.8	166.2	237.4	140.4	207.7	278.9	165.0	249.3	320.5	189.5	290.8	362.0	214.1
Guangdong	214.7	183.4	110.5	141.8	83.9	147.3	178.6	105.7	184.1	215.5	127.4	221.0	252.3	149.2	257.8	289.1	171.0
Guangxi	259.7	154.9	94.5	199.3	117.8	125.9	230.7	136.5	157.4	262.2	155.1	188.9	293.7	173.7	220.4	325.2	192.3
Hainan	257.3	179.7	112.3	189.9	112.3	149.8	227.4	134.5	187.2	264.8	156.6	224.7	302.3	178.8	262.1	339.7	200.9

Province	M-TII (μg/d)	M-SII (μg/d)	Iodine content 15 mg/kg			Iodine content 20 mg/kg			Iodine content 25 mg/kg			Iodine content 30 mg/kg			Iodine content 35 mg/kg		
			P-SII (μg/d)	P-TII (μg/d)	E-MUIC (μg/L)	P-SII (μg/d)	P-TII (μg/d)	E-MUIC (μg/L)	P-SII (μg/d)	P-TII (μg/d)	E-MUIC (μg/L)	P-SII (μg/d)	P-TII (μg/d)	E-MUIC (μg/L)	P-SII (μg/d)	P-TII (μg/d)	E-MUIC (μg/L)
Chongqing	327.2	187.0	107.4	247.7	146.5	143.3	283.5	167.7	179.1	319.3	188.8	214.9	355.1	210.0	250.7	390.9	231.2
Sichuan	289.0	198.1	109.2	200.1	118.4	145.7	236.5	139.9	182.1	272.9	161.4	218.5	309.4	183.0	254.9	345.8	204.5
Guizhou	338.5	207.3	116.9	248.1	146.7	155.9	287.1	169.8	194.9	326.0	192.8	233.8	365.0	215.9	272.8	404.0	238.9
Yunnan	299.3	187.4	115.6	227.6	134.6	154.2	266.1	157.4	192.7	304.7	180.2	231.3	343.2	203.0	269.8	381.8	225.8
Tibet	250.7	250.7	141.7	141.7	83.8	188.9	188.9	111.7	236.1	236.1	139.6	283.3	283.3	167.6	330.6	330.6	195.5
Shaanxi	373.5	197.4	120.4	296.5	175.3	160.5	336.6	199.1	200.6	376.7	222.8	240.7	416.8	246.5	280.8	456.9	270.3
Gansu	385.3	197.3	114.3	302.3	178.8	152.4	340.4	201.3	190.5	378.5	223.8	228.6	416.6	246.4	266.7	454.7	268.9
Qinghai	369.1	241.2	136.0	263.9	156.1	181.3	309.3	182.9	226.7	354.6	209.7	272.0	399.9	236.5	317.3	445.3	263.3
Ningxia	441.3	193.3	111.9	360.0	212.9	149.2	397.3	235.0	186.5	434.6	257.0	223.8	471.9	279.1	261.2	509.2	301.2
Xinjiang	258.5	249.4	136.0	145.2	85.9	181.4	190.5	112.7	226.7	235.9	139.5	272.1	281.2	166.3	317.4	326.5	193.1
Total	305.0	186.9	112.1	230.3	136.2	149.5	267.7	158.3	186.9	305.0	180.4	224.2	342.4	202.5	261.6	379.8	224.6

Note: M-TII represents the estimated total daily iodine intake from diet, drinking water and salt according to the latest MUIC;
M-SII represents the estimated daily salt iodine intake according to the latest salt consumption and the actual iodine content;
P-SII represents the estimated daily salt iodine intake of each province according to the latest salt consumption and the preset salt iodine content;
P-TII represents the estimated total daily iodine intake from diet, drinking water and salt according to the preset salt iodine content;
E-MUIC represents the estimated median of urinary iodine after the adjustment of salt iodine content.

Table S4. Estimated iodine intake and urine iodine levels after changes in salt iodine content in 31 provinces of China (pregnant women).

Province	M-TII (μg/d)	M-SII (μg/d)	Iodine content 25 mg/kg			Iodine content 30 mg/kg			Iodine content 35 mg/kg			Iodine content 40 mg/kg		
			P-SII (μg/d)	P-TII (μg/d)	E-MUIC (μg/L)	P-SII (μg/d)	P-TII (μg/d)	E-MUIC (μg/L)	P-SII (μg/d)	P-TII (μg/d)	E-MUIC (μg/L)	P-SII (μg/d)	P-TII (μg/d)	E-MUIC (μg/L)
Beijing	252.3	203.8	208.8	257.2	152.1	250.6	299.1	176.9	292.4	340.8	201.6	334.1	382.6	226.3
Tianjin	278.8	177.1	178.5	280.2	165.7	214.2	315.9	186.9	249.9	351.6	208.0	285.6	387.4	229.1
Hebei	295.2	177.0	190.0	308.2	182.3	227.9	346.1	204.7	265.9	384.1	227.2	303.9	422.1	249.6
Shanxi	322.1	182.5	191.7	331.3	195.9	230.0	369.6	218.6	268.4	408.0	241.3	306.7	446.3	264.0
Inner Mongolia	298.1	171.5	184.1	310.7	183.7	220.9	347.4	205.5	257.7	384.2	227.2	294.5	421.0	249.0
Liaoning	256.3	147.6	151.8	260.6	154.1	182.2	290.9	172.1	212.6	321.3	190.0	242.9	351.7	208.0
Jilin	284.2	151.1	156.8	289.9	171.4	188.1	321.2	190.0	219.5	352.6	208.5	250.8	383.9	227.1
Heilongjiang	319.1	155.9	155.9	319.0	188.7	187.1	350.2	207.1	218.3	381.4	225.6	249.5	412.6	244.0
Shanghai	241.6	152.4	158.0	247.3	146.3	189.7	278.9	165.0	221.3	310.5	183.7	252.9	342.1	202.3
Jiangsu	277.0	170.4	178.9	285.5	168.9	214.7	321.3	190.0	250.5	357.1	211.2	286.3	392.9	232.4
Zhejiang	232.7	163.2	172.9	242.4	143.3	207.4	276.9	163.8	242.0	311.5	184.2	276.6	346.0	204.7
Anhui	330.4	187.0	194.8	338.2	200.0	233.8	377.1	223.1	272.7	416.1	246.1	311.7	455.1	269.1
Fujian	242.8	157.2	161.1	246.7	145.9	193.3	278.9	164.9	225.5	311.1	184.0	257.8	343.3	203.1
Jiangxi	320.7	178.7	181.6	323.6	191.4	217.9	360.0	212.9	254.3	396.3	234.4	290.6	432.6	255.9
Shandong	271.9	159.7	169.9	282.1	166.8	203.8	316.1	186.9	237.8	350.0	207.0	271.8	384.0	227.1
Henan	346.8	231.4	220.8	336.2	198.8	264.9	380.3	224.9	309.1	424.5	251.1	353.2	468.7	277.2
Hubei	297.4	225.7	223.9	295.6	174.9	268.7	340.4	201.3	313.5	385.2	227.8	358.3	430.0	254.3
Hunan	337.0	222.7	207.7	322.0	190.5	249.3	363.6	215.0	290.8	405.1	239.6	332.4	446.7	264.2
Guangdong	290.7	183.4	184.1	291.3	172.3	221.0	328.2	194.1	257.8	365.0	215.9	294.6	401.9	237.7
Guangxi	305.4	154.9	157.4	307.9	182.1	188.9	339.4	200.7	220.4	370.9	219.3	251.9	402.3	238.0

Province	M-TII (μg/d)	M-SII (μg/d)	Iodine content 25 mg/kg			Iodine content 30 mg/kg			Iodine content 35 mg/kg			Iodine content 40 mg/kg		
			P-SII (μg/d)	P-TII (μg/d)	E-MUIC (μg/L)	P-SII (μg/d)	P-TII (μg/d)	E-MUIC (μg/L)	P-SII (μg/d)	P-TII (μg/d)	E-MUIC (μg/L)	P-SII (μg/d)	P-TII (μg/d)	E-MUIC (μg/L)
Hainan	221.7	179.7	187.2	229.1	135.5	224.7	266.6	157.7	262.1	304.0	179.8	299.6	341.5	202.0
Chongqing	303.7	187.0	179.1	295.8	174.9	214.9	331.6	196.1	250.7	367.4	217.3	286.5	403.2	238.5
Sichuan	303.2	198.1	182.1	287.2	169.8	218.5	323.6	191.4	254.9	360.0	212.9	291.3	396.4	234.4
Guizhou	294.2	207.3	194.9	281.8	166.6	233.8	320.7	189.7	272.8	359.7	212.7	311.8	398.6	235.8
Yunnan	291.7	187.4	192.7	297.0	175.7	231.3	335.6	198.5	269.8	374.2	221.3	308.4	412.7	244.1
Tibet	266.3	266.3	236.1	236.1	139.6	283.3	283.3	167.6	330.6	330.6	195.5	377.8	377.8	223.4
Shannxi	312.3	197.4	200.6	315.5	186.6	240.7	355.6	210.3	280.8	395.7	234.1	320.9	435.9	257.8
Gansu	302.1	197.3	190.5	295.3	174.6	228.6	333.4	197.2	266.7	371.5	219.7	304.8	409.6	242.2
Qinghai	312.5	241.2	226.7	298.0	176.2	272.0	343.3	203.0	317.3	388.6	229.8	362.7	434.0	256.7
Ningxia	299.8	193.3	186.5	293.0	173.3	223.8	330.4	195.4	261.2	367.7	217.5	298.5	405.0	239.5
Xinjiang	350.7	249.4	226.7	328.0	194.0	272.1	373.3	220.8	317.4	418.7	247.6	362.7	464.0	274.4
Total	296.7	186.9	186.9	296.8	175.5	224.2	334.1	197.6	261.6	371.5	219.7	299.0	408.9	241.8

Note: M-TII represents the estimated total daily iodine intake from diet, drinking water and salt according to the latest MUIC;
M-SII represents the estimated daily salt iodine intake according to the latest salt consumption and the actual iodine content;
P-SII represents the estimated daily salt iodine intake of each province according to the latest salt consumption and the actual iodine content;
P-TII represents the estimated total daily iodine intake from diet, drinking water and salt according to the preset salt iodine content;
E-MUIC represents the estimated median of urinary iodine after the adjustment of salt iodine content.
